# Emotion Processing in Parkinson’s Disease: A Three-Level Study on Recognition, Representation, and Regulation

**DOI:** 10.1371/journal.pone.0131470

**Published:** 2015-06-25

**Authors:** Ivan Enrici, Mauro Adenzato, Rita B. Ardito, Antonia Mitkova, Marco Cavallo, Maurizio Zibetti, Leonardo Lopiano, Lorys Castelli

**Affiliations:** 1 Department of Philosophy and Educational Sciences, University of Turin, Turin, Italy; 2 Center for Cognitive Science, University of Turin, Turin, Italy; 3 Neuroscience Institute of Turin, Turin, Italy; 4 Department of Psychology, University of Turin, Turin, Italy; 5 eCampus University, Novedrate, Como, Italy; 6 Azienda Sanitaria Locale Torino 3, Turin, Italy; 7 Department of Neuroscience, University of Turin, Turin, Italy; Tilburg University, NETHERLANDS

## Abstract

**Background:**

Parkinson’s disease (PD) is characterised by well-known motor symptoms, whereas the presence of cognitive non-motor symptoms, such as emotional disturbances, is still underestimated. One of the major problems in studying emotion deficits in PD is an atomising approach that does not take into account different levels of emotion elaboration. Our study addressed the question of whether people with PD exhibit difficulties in one or more specific dimensions of emotion processing, investigating three different levels of analyses, that is, recognition, representation, and regulation.

**Methodology:**

Thirty-two consecutive medicated patients with PD and 25 healthy controls were enrolled in the study. Participants performed a three-level analysis assessment of emotional processing using quantitative standardised emotional tasks: the Ekman 60-Faces for emotion recognition, the full 36-item version of the Reading the Mind in the Eyes (RME) for emotion representation, and the 20-item Toronto Alexithymia Scale (TAS-20) for emotion regulation.

**Principal Findings:**

Regarding emotion recognition, patients obtained significantly worse scores than controls in the total score of Ekman 60-Faces but not in any other basic emotions. For emotion representation, patients obtained significantly worse scores than controls in the RME experimental score but no in the RME gender control task. Finally, on emotion regulation, PD and controls did not perform differently at TAS-20 and no specific differences were found on TAS-20 subscales. The PD impairments on emotion recognition and representation do not correlate with dopamine therapy, disease severity, or with the duration of illness. These results are independent from other cognitive processes, such as global cognitive status and executive function, or from psychiatric status, such as depression, anxiety or apathy.

**Conclusions:**

These results may contribute to better understanding of the emotional problems that are often seen in patients with PD and the measures used to test these problems, in particular on the use of different versions of the RME task.

## Introduction

Parkinson’s disease (PD) is a common neurodegenerative condition associated with the loss of dopamine producing neurons in the pars compacta region of the substantia nigra [1]. Main motor manifestations of PD include resting tremor, bradykinesia, stiffness (rigidity), soft voice, micrographia, shuffling steps, and difficulties with balance. Although the motor symptoms are well known, this condition is also associated with a spectrum of non-motor dysfunctions, such as cognitive impairment (mainly memory and executive functions) and neuropsychiatric disturbances (mainly depression, anxiety, apathy, and emotional dysregulation disturbances), symptoms that can be just as disabling as motor dysfunctions [2].

One of the key dimensions recently investigated among the social cognitive processes affected in PD is the ability to manage emotional information in order to understand and interpret social situations properly [3–6]. Even though the studies are inconclusive on the definition of the degree and selectivity of impairments, there is evidence pointing to the existence of emotional disorders in three main domains: emotion recognition, emotion representations, and emotion regulation. For example, patients with PD show specific deficits in the recognition of emotional facial expression, in particular the disgust expression, frequently accompanied by impairment in the recognition of fear facial expressions ([7], for a review). In addition, different studies highlight specific impairment of emotion representation, such as those associated with affective Theory of Mind (ToM), i.e., the ability to represent others’ affective mental states such as belief about feelings ([6], for a review). Lastly, further studies showed reduced reactivity to emotional stimuli (i.e., emotional blunting), and linked these deficits to the inability to identify and describe one’s feelings distinguishing between feelings and bodily sensations of emotional arousal, clinically defined as alexithymia [8–10]. Although there appears to be some convergence of results, several discrepancies regarding emotion processing in PD have been described (e.g., [5, 11, 12]), and further studies are needed to clarify the emotional profile of patients with PD.

One of the major problems in studying emotion deficits in a neurodegenerative population is an atomising approach that does not take into account different levels of emotion elaboration [13–15]. In addition, the terminology used by different authors in the domain of social cognitive processes is sometimes confusing. For example, the lack of agreement around the definitions of emotion perception and ToM has been recently described [16]. A useful model for testing emotional deficits was proposed by Decety in the domain of human empathy [17]. According to this model empathy involves at least two main areas, each with different developmental trajectories: emotion understanding, i.e., the ability to recognise and represent emotions, and emotion regulation, i.e., the ability to control emotion, affect, and motivation. In line with this proposal, in the present study we investigate emotion understanding–distinguishing between emotion recognition and representation–, and emotion regulation. Whereas emotion recognition is regarded as a low-level perceptual process involved in the identification of emotionally salient information in the environment, emotion representation concerns a ToM higher-level reasoning process involved in the integration and inference of social information (both emotional and intentional) [18–20]. In particular, according to this perspective basic emotions are recognised cross-culturally and are situation-based while social emotions are belief-based and depend on other people’s feelings or thoughts. In other words, the more complex the emotion, the greater degree of ToM is required to decode it [16, 18].

In line with these considerations, a crucial question concerning emotion processing in PD is whether emotional disorders can be specifically explained by a deficit in the identification of a specific emotion (emotion recognition), in the attribution of a specific affective mental state (emotion representation), or by a deficit in the management of one’s emotion (emotion regulation). Although current studies have investigated single specific dimensions of emotional disorder in PD, no study has yet compared these different dimensions of emotional processing in the same experimental framework. The present research investigates PD emotional processing comparing the three different levels: emotion recognition, representation, and regulation. Accordingly, we used three quantitative standardised emotional tasks to test emotional processing: (1) the Ekman 60-Faces task to test the recognition of basic facial emotions [21]; (2) the Reading the Mind in the Eyes (RME) test, an affective ToM task based on the implicit ability to represent the feelings of another person by observing only their eyes [22]; and (3) the 20-item Toronto Alexithymia Scale (TAS-20), a validated self-report scale assessing the three emotion regulation facets, namely the difficulty in identifying feelings, difficulty in describing feelings, and externally oriented thinking [23].

## Materials and Methods

### Ethics Statement

The study was approved by the San Giovanni Battista University Hospital’s ethics committee and was conducted in accordance with the Declaration of Helsinki. All the participants gave their written informed consent to participate in the study.

### Participants

Thirty-two consecutive patients with PD under dopaminergic replacement therapy and 25 healthy controls (HC) with negative neurological and psychiatric history were enrolled in the study. All patients had been referred consecutively to the Neurology Unit of the Turin University Hospital for their standard care visits. Inclusion criteria for the patients included a diagnosis of idiopathic Parkinson’s disease, undergoing dopaminergic pharmacological treatment, and an age greater or equal to 40 years. Exclusion criteria for all subjects were the presence of dementia or severe cognitive impairment (Mini Mental State Examination, MMSE ≤ 26), and presence of other neurological or psychiatric disorders, such as severe depression. All patients were scored on the Unified Parkinson’s Disease Rating Scale (UPDRS) I to IV [24] and Hoehn and Yahr Staging Scale (H&Y) [25]. Depression severity was assessed using the Beck Depression Inventory (BDI) [26], a validated measure for depression symptoms in PD [27]. All patients included in the study were administered their daily optimal dopamine replacement therapy (Levodopa preparations, and/or dopamine receptor agonists). Patients were assessed for neuropsychological and psychological profile in their usual optimal medication-on condition (MED ON).

### Neuropsychological and clinical assessment

The MMSE [28] and Frontal Assessment Battery (FAB) [29] were administered to all subjects for a preliminary screening of global cognitive and executive functioning. Patients were given a standardised neuropsychological test battery in order to assess reasoning, memory, and attentional executive functions [30]. Visuospatial abilities and reasoning was evaluated by means of the Attentional Matrices task [31] and the Raven Color Matrices (PM 47) [32]. Verbal and spatial short-term memory was assessed by means of the bisyllabic word repetition test (BWR) [31] and Corsi’s block-tapping test [31], respectively. The assessment of verbal learning was achieved by means of the paired-associate learning (PAL) [33], a Wechsler Memory Scale subtest. Frontal lobe executive functions, including the development of abstract concepts and the shift of attention and motor sets, were assessed by means of the Trail Making Test Part B [34] and the Nelson Modified Card Sorting Test (MCST) [35], a modified version of the Wisconsin Card Sorting Test. In addition, patients were given phonemic [36] and category verbal fluency tasks [31]. The clinical assessment included the Apathy Evaluation Scale [37], State Trait Anxiety Inventory (STAI) [38], in particular, (STAI-X1, state anxiety) and (STAI-X2, trait anxiety), as well as BDI.

### Emotional assessment

Patients with PD and HC performed a three-level analysis assessment of emotional processing using quantitative standardised emotional tasks: the Ekman 60-Faces task for emotion recognition, the 36-item full version RME task for emotion representation, and the TAS-20 for emotion regulation.

The Ekman 60-Faces task is a tool to test the recognition of basic facial emotions using photographs of the faces of 10 people (6 women), selected from Ekman and Friesen [21]. The Italian version of this task was used [39]. Each photograph displayed six facial expressions corresponding to six emotions: anger, disgust, fear, sadness, happiness, and surprise, giving 60 photographs (10 for each emotion). Pictures were presented on a computer screen one at a time in pseudo-random order and participants were asked to select one of six emotion labels (anger, disgust, fear, sadness, happiness, surprise) that best described the facial expression shown. No feedback was given as to the appropriateness of any response.

The 36-item full version of the RME task is an advanced affective ToM task involving presentation of photographs of the eye region of human faces, and is based on the ability to represent the feelings of another person by observing only their eyes [22]. Participants are required to choose which word, among four options, best describes what the character in the photograph is thinking or feeling (e.g., which of the following words best describes the eye region shown: excited, relieved, shy, or despondent). Explanations of emotion labels, according to the definitions provided by the Italian version of the task [40], were given to subjects when asked by the subjects themselves. The total number of correct choices indicates the RME performance. In addition to the RME, we administered the RME gender control task (identification of the gender of the character in the photograph).

The 20-item Toronto Alexithymia Scale is an extensively validated self-report questionnaire to assess emotion regulation [23, 41]. The scale comprises three subscales that investigate the following factors: (F1) difficulty in identifying feelings (e.g., “I am often confused about what emotion I am feeling”; “I have feelings that I cannot quite identify”); (F2) difficulty in describing and communicating feelings (e.g., “I find it hard to describe how I feel about people”; “It is difficult for me to reveal my innermost feelings, even to close friends”); (F3) externally oriented thinking (e.g., “I prefer to analyse problems rather than just describe them”; “I prefer talking to people about their daily activities rather than their feelings”). The total score on the questionnaire allowed categorizing the subjects according to their alexithymic dimension: non-alexithymic (score: 20–51), borderline alexithymic (score: 52–60), or alexithymic (score ≥61). The Italian version of this test was used [42].

### Statistical analysis

The statistical analyses were carried out with SPSS version 21.0 for Windows. For normally distributed data, parametric tests were used (t-test for independent samples, and repeated-measures analysis of variance, ANOVA). In cases of significant deviations from the assumption of normality of variables distribution, we used non-parametric methods (Mann–Whitney U-test), as specified in the related tables. Besides, to investigate the correlations between variables in the groups of participants, we ran bivariate Pearson’s correlations. The level of significance for all statistical tests was set at p < 0.05.

## Results

### Demographic and preliminary clinical assessments

Table 1 shows the demographic, neuropsychological, and clinical data of PD and HC. Patients with PD and HC were well matched for demographic variables such as age, education, and gender. Regarding the neuropsychological measures, patients and HC did not differ in terms of their MMSE score, whereas the two groups differed in the FAB scores; however, only two patients obtained a FAB score below the cut-off of 15.

**Table 1:** Demographic, neuropsychological and clinical measures.

	PD patients (N = 32)	HC (N = 25)	t-test or other statistics (specified)
*Demographic variables*			
Age (years)	57.97 (7.20)	56.32 (6.35)	0.902 NS
Education (years)	9.47 (3.95)	10.80 (3.83)	1.279 NS
Gender—M:F	17:15	11:14	*Χ* ^2^ = 0.468 NS
*Neuropsychological measures*			
MMSE	28.78 (1.09)	29.20 (1.08)	1.467 NS
FAB	16.74 (1.30)	17.42 (0.85)	2.280 p = 0.027
Corsi’s Block-Tapping Test	4.72 (0.66)	-	-
Attentional Matrices	47.11 (6.74)	-	-
Raven Color Matrices	30.64 (3.91)	-	-
Bisyllabic Word Repetition Test	4.01 (0.70)	-	-
Paired-Associate Learning	13.24 (2.75)	-	-
Trail Making Test Part B	103.13 (111.15)	-	-
Nelson Modified Card Sorting Test		-	-
Categories	5.81 (0.48)	-	-
Errors	4.84 (3.75)	-	-
Perseverations	2.00 (2.07)	-	-
Phonemic verbal fluency	38.61 (8.10)	-	-
Category verbal fluency	21.28 (3.77)	-	-
*Clinical variables*	13.21 (6.11)	-	-
BDI	13.21 (6.11)	-	-
STAI-X1	44.97 (10.17)	-	-
STAI-X2	44.66 (8.89)	-	-
Apathy Evaluation Scale	13.45 (4.99)	-	-
Duration of illness (years)	10.56 (3.88)	-	-
Ledd	1075.33 (395.37)	-	-
UPDRS III	33.69 (9.26)	-	-
H&Y Off	2.81 (0.86)	-	-
H&Y On	1.60 (0.67)	-	-

BDI = Beck Depression Inventory; FAB = Frontal Assessment Battery; H&Y = Hoehn and Yahr; HC = Healthy Controls; Ledd = Levodopa equivalent daily dose; MMSE = Mini Mental State Examination; PD = Parkinson’s Disease; STAI = State Trait Anxiety Inventory; UPDRS = Unified Parkinson’s Disease Rating Scale.

### Emotional assessment

The difference in performance between patients with PD and HC for the TAS-20, RME, and Ekman 60-Faces tests are presented in Table 2. Patients obtained significantly worse scores than HC in the RME experimental score, and in the total score of Ekman 60-Faces test. For comparisons on basic emotions between the two groups of participants (sadness, happiness, anger, disgust, fear, surprise), we adopted a prudent and conservative approach by using Bonferroni’s correction from multiple comparisons (α = 0.05/6 = 0.008). Accordingly, none of the comparisons between the two groups reached statistical significance. Taking into account the possible influence of the frontal measure FAB on these group comparisons, we repeated them by considering the FAB score as a covariate, and no significant change emerged. Moreover, no statistically significant differences were found between the two groups in the RME gender control task score and TAS-20 total score. In order to investigate the possible relationship between emotional (TAS-20 total score, RME and Ekman 60-Faces) and psychiatric (BDI, STAI, and Apathy Evaluation Scale) measures, we analysed bivariate correlations in the group of patients with PD between all these variables, but did not find any statistically significant differences, with the only exception of the significant correlation between TAS-20 total score and STAI-X2 (r = 0.387, p = 0.046).

**Table 2:** Emotion processing measures.

	PD patients (N = 32)	HC (N = 25)	t-test or Mann-Whitney U test
*Emotion recognition*			
Ekman-total	43.75 (5.80)	48.12 (5.74)	2.806 p = 0.007
Ekman-sadness	6.59 (1.95)	7.42 (1.59)	U = 281.5 p = 0.084 NS
Ekman-happiness	9.72 (0.73)	9.71 (0.75)	U = 380.5 p = 0.928 NS
Ekman-anger	6.66 (2.19)	8.04 (1.78)	U = 243.5 p = 0.018 NS
Ekman-disgust	7.84 (1.94)	8.58 (1.86)	U = 284.5 p = 0.091 NS
Ekman-fear	4.25 (1.97)	5.33 (2.57)	U = 277.7 p = 0.072 NS
Ekman-surprise	8.69 (1.42)	9.04 (1.16)	U = 331 p = 0.357 NS
*Emotion representation*			
RME	20.59 (5.14)	23.40 (4.65)	2.130 p = 0.038
RME control	34.09 (2.20)	35.04 (1.14)	1.950 NS
*Emotion regulation*			
TAS 20	53.77 (10.72)	50.96 (13.28)	0.868 NS
TAS 20-F1	18.25 (6.26)	16.55 (6.61)	0.907 NS
TAS 20-F2	15.54 (4.19)	13.75 (4.74)	1.377 NS
TAS 20-F3	20.75 (5.09)	20.15 (5.39)	0.393 NS

HC = Healthy Controls; PD = Parkinson’s Disease; RME = Reading the Mind in the Eyes; TAS-20 = Twenty-item Toronto Alexithymia Scale.

Alexithymia total scores ranged from 34 to 70 in the PD group (median = 56.50) and from 24 to 76 in the HC group (median = 50). Based on the TAS-20 score, 9 patients with PD (30%) and 7 HC (28%) could be classified as alexithymic; 13 patients with PD (43.33%) and 13 HC (52%) were non-alexithymic, and the remaining 8 patients with PD (26.67%) and 5 HC (20%) obtained borderline scores. Chi-square analysis comparing the number of individuals with and without alexithymia (non-alexithymic and borderline scores) in the two groups (PD and HC) showed no statistically significant difference (Chi-square = 0.026, p = 0.871).

In addition, bivariate correlations were calculated for the four categories of subjects (PD and HC with and without alexithymia) and the emotional and psychiatric measures administered: patients with PD and alexithymia showed a significant correlation between TAS-20 and Ekman disgust scores, RME and Ekman total scores, RME and Ekman anger scores, and BDI and STAI-X2 scores. Patients with PD without alexithymia showed a significant correlation between TAS-20 and STAI-X2 scores, RME and Ekman total scores, RME and Ekman anger scores, RME and fear scores, and BDI and STAI-X1 scores. HC with alexithymia showed a significant correlation between RME and Ekman sadness scores, whereas HC without alexithymia showed a significant correlation between RME and Ekman total scores, and RME and Ekman anger scores.

In the overall group of patients with PD, as well as in the subgroups with or without alexithymia, we investigated the possible correlation between emotional measures (TAS-20, RME, Ekman 60-Faces) and, respectively, the Levodopa equivalent daily dose (Ledd) and duration of illness. No statistically significant correlations emerged. Furthermore, in patients with PD, we investigated the correlation between the performance on emotional measures and, respectively, the UPDRS III and the H&Y scores (indicators of severity of illness in medication-off condition), and any significance correlations emerged. Lastly, for explorative purpose, we investigated the correlations between performance on the Ekman 60-Faces tests, RME, and TAS-20 (total score and subscales score) and the measures of visuospatial abilities (i.e., attentional matrices, and Corsi’s Block-Tapping Test), the attentional matrices score correlated significantly with RME (r = 0.551, p = 0.001), Ekman total score (r = 0.415, p = 0.020), and anger (r = 0.431, p = 0.015), whereas Corsi’s Block-Tapping Test score correlated significantly with disgust (r = 0.477, p = 0.007).

## Discussion

Summing up, results of the present study show a specific emotion-representation deficit in the PD sample, an overall impairment in emotion recognition, and no specific impairment in emotion regulation. Patients with PD and HC were well matched in age, level of education, and gender. Besides, they did not differ in their performance in the MMSE. Though, a significant difference between the two groups in the FAB scores emerged, with patients performing worse than controls. However, regarding this comparison, it is worth noting that only two patients out of 32 got a score lower than the clinical cut-off score (i.e., 15/18), strongly suggesting that this inter-group difference was only statistical but not clinical. Regarding emotion recognition, patients obtained significantly worse scores than HC in the total score of Ekman 60-Faces test but the differences in the recognition of each specific basic emotion was not significant. For emotion representation, patients obtained significantly worse scores than HC in the RME experimental score but no in the RME gender control task. Finally, regarding emotion regulation, PD and HC did not perform differently at TAS-20 and no specific differences were found on TAS-20 subscales. The PD impairments on emotion recognition and representation do not correlate with dopamine therapy, disease severity, or with the duration of illness. These results are independent from other cognitive processes, such as global cognitive status and executive function (MMSE and FAB), or from psychiatric status such as depression, anxiety or apathy. Furthermore, as there are no statistically significant difference between patients with PD and HC on the recognition of specific basic emotions and on the RME gender control task, we can exclude that our results to the experimental version of the RME are due to a specific deficit in paying attention to the eye region of the face, a possibility that can not be excluded in principle [43–45].

### Emotion recognition

To the best of our knowledge, at least five studies investigated emotion recognition level using the Ekman 60-Faces task in a PD population [46–50]. Sprengelmeyer and colleagues [50] showed evidence for impaired recognition of emotional facial expressions in patients with PD than in HC. These deficits were more consistent in the non-medicated than in medicated patients, specifically in recognising disgust and anger. A selective impairment in the recognition of anger was presented by Lawrence and colleagues [48] in a group of non-medicated patients with PD. Treatment with dopamine replacement therapy (DRT) could mask deficits present in PD; therefore, the study assessed facial expression recognition in a group of patients with PD transiently withdrawn from DRT. The impairment on anger was not related to the overall disease severity or to depression symptoms, and with relatively sparse recognition of other emotions and facial identity processing. Different results were reported by Ibarretxe-Bilbao and colleagues [47], where patients with early PD obtained lower scores than HC in all emotions except happiness, in which a ceiling-effect was observed for both PD and HC groups. Moreover, using structural magnetic resonance to test structural changes between the groups in the orbitofrontal cortex and amygdala, the study found grey matter loss in the orbitofrontal cortex bilaterally and in the right amygdala, but only the former presented a strong correlation with the performance on emotion recognition. A positive correlation between individual emotion recognition and grey matter volume was found by Baggio and colleagues [46] using a voxel-based morphometry analysis, particularly for negative emotion. Correlation was found among the right orbitofrontal cortex, amygdala and postcentral gyrus and sadness identification, the right occipital fusiform gyrus, ventral striatum and subgenual cortex and anger identification, and the anterior cingulate cortex and disgust identification. In the Ekman 60-Faces task performance, PD patients presented with impaired recognition of all the negative emotions assessed, whereas no differences were observed for the recognition of surprise and happiness. Finally, a recent study conducted by Ricciardi and colleagues [49], that use both Ekman 60-Faces task and TAS-20, showed that patients with PD performed significantly worse than HC in recognising surprise facial expression, with no difference in alexithymia. Interestingly, using a dynamic expression video recording protocol, the study reported a significant impairment in producing global facial expression in patients with PD than in HC. The results revealed both a negative correlation between the factor F3 of TAS-20 (externally orientated thinking) and the patient’s capability to express disgust, as well as a positive correlation between the Ekman 60-Faces total score and the patient’s capability to express disgust.

Taking together, the studies that use the Ekman 60-Faces reported a general impairment in emotion recognition in PD without a specific agreement on particular emotion expression impairment. Our results are in line with these data: our PD sample obtained significantly worse scores than HC in the total score of Ekman 60-Faces test but with no specific impairment on any single basic emotion.

### Emotion representation

Recently, an advanced view for the representational complex ability we refer to as ToM (the general ability to represents other mental state [51–56]) has been suggested depending on the nature of the mental state that is represented, such as the affective ToM subcomponent, i.e., representation of belief about feelings, and cognitive ToM subcomponent, i.e., representation of belief about belief [57–59]. Although cognitive ToM difficulties in PD are clearly involved in the early clinical stages, findings on affective ToM are debatable. The spatiotemporal progression of striatal dopamine depletion in PD supports the hypothesis that this component could be impaired in more advanced stages, when the ventromedial prefrontal cortex—found to be involved in affective ToM—is hypostimulated by the orbital frontostriatal loop [6]. Although the RME task is frequently used to assess emotion representation in PD, there is no agreement in current literature as to whether or not RME performance is impaired in patients with PD, and findings on affective ToM impairment in different clinical stages of PD are still controversial [3, 6, 58].

To the best of our knowledge, only four studies showed a preserved performance on the RME task by medicated patients in the early [60, 61] and moderate stages of PD [60, 62, 63] with a mean disease duration from 1.69 years in Roca et al. [61], up to 10.2 years in Péron et al. [63] (see Table 3). (Excluding severe conditions of PD, i.e. H&Y stages 4 and 5, according to the MDS Task Force Guidelines on the Hoehn and Yahr Scale [64], clinical stages can be defined as early on H&Y stages 1, 1.5 and 2, and moderate on H&Y stage 2.5 and 3).

**Table 3:** RME task versions used to test emotion representation in PD.

Authors	Sample	PD mean age in years (SD)	Disease duration in years (SD)	Hoen and Yahr stage	RME items	RME performance
Euteneuer et al., 2009	21PD/23HC	67.6 (7.3)	7.1 (6)	2.5	36/36	Preserved
Péron et al., 2009	17 early PD/26HC 27 advanced PD/ 26HC	61.0 (7.1) 56.6 (7.8)	2.5 (1.5) 10.2 (4.9)	Off 1.5 (0.7)/On 1.0 (0.9) Off 2.5 (1.0)/On 1.3 (0.9)	17/36	Preserved
Péron et al., 2010 *	13PD/13HC	53.3 (8.5)	10.5 (3.6)	Off 2.3 (0.8)/On 1.2 (0.6)	17/36	Preserved
Roca et al., 2010 **	16PD/35HC	63.4 (8.47)	1.69 (1.55)	1.42 (0.57)	15/36	Preserved
Mimura et al., 2006	18PD/40HC	68.9 (7.0)	N/A	II/III	25/36	Impaired
Bodden et al., 2010	21PD/21 HC	63.7 (10.0)	5.1 (2.8)	2.5 (Range 1.0–3.0)	36/36	Impaired
Tsuruya et al., 2011	20PD/20HC	70.5 (8.6)	5.1 (0.7)	1.5 (0.7)	20/36	Impaired
McKinlay et al., 2013	50PD/49HC	66.34 (6.71)	N/A	2.26 (0.82) Males 2.08 (0.84) Females	36/36	Impaired
Poletti et al., 2013	35PD/35HC	66.97 (11.79)	6.31	2.08 (0.72)	36/36	Impaired
Xi et al. 2014	15PD/15HC	60.73 (11.79)	4.33 (5.05)	1.97 (0.67)	34/36	Impaired
Present study	32PD/25HC	57.97 (7.20)	10.56 (3.88)	On 1.6(0.67)/Off 2.81 (0.86)	36/36	Impaired

HC = Healthy Controls; PD = Parkinson’s Disease; RME = Reading the Mind in the Eyes;

* Data of preoperative patients

** Data of medicated patients

A study conducted by Roca and colleagues [61] showed preserved performance on the RME task in a group of non-medicated de novo patients with PD compared to a group of medicated patients with PD and HC. A similar result was found by Péron and colleagues [63] investigating the effects of surgery where no differences were reported in RME task results between pre-operative patients with PD (prior to deep brain stimulation, DBS) and HC, whereas post-operative patients with PD had lower performance than HC and pre-operative patients.

Conversely, six studies reported lower RME performances of medicated patients with PD in comparison to HC [65–70]. Mimura and colleagues [67] reported that medicated patients at early to moderate PD stages (Range of H&Y Stage 1–3) had poorer scores than HC on RME, even though the authors highlighted that RME performances by both PD and HC groups were relatively high. In a study by Tsuruya and colleagues [69], patients had a mean PD duration of 5.1 ± 0.7 years and were in early stages (H&Y Mean Stage 1.5 ± 0.7). Bodden and colleagues [65] reported that patients had a mean PD duration of 5.1 ± 2.8 years but the clinical staging of PD was heterogeneous, with both early and moderate PD (H&Y Mean Stage 2.5, Range 1–3). Interestingly, Tsuruya and colleagues [69] reported an impaired performance of emotion representation in patients with early PD; however, they performed as well as HC in the semantic discrimination and gender (control) attribution tasks. Therefore, the impaired ability to infer the emotional states of others found in patients with PD was not due to a failure in discriminating emotional adjectives (semantic discrimination) or perceptual problems in visually analysing the eye-gaze region (gender attribution).

Two recent studies seem to confirm the trend toward impairment in emotion representation in both early and moderate PD patients [68, 70]. Using a full 36-item version of RME, Poletti and colleagues [68] found that patients at both early and moderate PD stages (with at least 6.31 ± 4.02 years of disease duration and H&Y mean 2.08 ± 0.72) were impaired in comparison to HC. Interestingly, patients with moderate PD had poorer RME performances than those with early PD, but this difference did not reach statistical significance when variables correlating with RME were controlled for. Similar results were found in a very recent study that used a 34-item version of RME on patients at early PD stages [70]. Results showed that patients with PD had a specific impairment of emotion representation in comparison to HC, at a very early stage of PD progression (4.33 ± 5.05 years of disease duration and H&Y mean 1.97 ± 0.67). These findings confirmed that emotion representation might be impaired in early PD.

Our results are in agreement with these latter studies. Using a full 36-item version of RME, we found a specific impairment on emotion representation in patients at an early to moderate stage of PD progression, with a mean disease duration of 10.56 ± 3.88 years and mean H&Y scores of 2.81 ± 0.86 (MED OFF). It is interesting to note that in most the previous studies [65, 68–70], the HC mean age was lower than that of the PD group (58.7 vs. 63.7 years, 66.97 vs. 68.45 years and 67.7 vs. 70.5 years, and 56.33 vs. 60.73 years, respectively). In our experimental sample, the mean age of PD and HC groups was 57.97 and 56.32 years, respectively, with no statistical difference between the two groups. This is particularly important, since RME performance has been reported to decline with age in healthy subjects [71].

Converging evidence on emotion representation deficits in patients with early PD was recently reported by Santangelo and colleagues [72] using a novel emotion attribution task to investigate the ability to attribute emotional state to others. The authors used short stories describing emotional situations designed to elicit attributions of sadness, fear, embarrassment, disgust, happiness, anger, and envy, and found emotion representation impairment in patients at very early stages of PD progression (6.8 ± 4.7 years of disease duration and H&Y mean 1.7 ± 0.6).

These results suggest that both affective and cognitive representations of the mental state could be simultaneously impaired in early PD (see also [6]); they confirm that the deficits in the two subcomponents of mental state representation (i.e., cognitive and affective ToM) may be linked to dysfunction of different frontosubcortical circuitries in early stages of PD [58].

One possible explanation for the discrepancy in PD performances found on emotion representation could be due to differences among the RME task versions used in the experimental setting (see Table 3). Almost all the studies that found deficits in the RME, including ours, adopted the full 36-item version of the task [65, 66, 68] or a version with majority of the items (25 items in Mimura et al. [67], 20 items in Tsuruya et al. [69] and 34 in Xi et al. [70]); however, almost all studies that found preserved performance in the RME adopted a shorter version (e.g., 17 items in Péron et al. [60, 63], and 15 items in Roca et al. [61]). Our results are in line with Poletti et al. [68]’ suggestion: the use of a shorter version of RME instead of the complete version could prevent detection of mild or sub-threshold impairments in affective ToM.

### Emotion regulation

Different studies used the TAS-20 to test emotion regulation in PD [8–10, 73–78]. One of the first studies that investigated emotion regulation in PD without a HC group [75], categorised 20.7% of their sample of patients with PD as alexithymic, 22.4% as borderline alexithymic, and 56.9% as non-alexithymic. They reported a strong correlation between alexithymia and severity of depression in this population. High scores obtained on the BDI were found to strongly predict high level of alexithymia in these patients. In a further study with a HC group, Costa and colleague [10] found that the prevalence of alexithymia was about double in patients with PD than in the HC group: 21.4% of patients with PD and 10.0% of controls could be classified as alexithymic, and the two groups significantly differed on global levels of alexithymia. Analyses of individual TAS-20 subscales showed a significant difference between groups only in the difficulty in describing and communicating feelings (F2), but not in identifying feelings (F1) and externally oriented thinking (F3). Lastly, their data showed significant associations among the BDI, STAI, and TAS-20 factors in both groups, and confirmed the previously reported high association between depression, anxiety, and alexithymia. A similar distribution pattern between PD and HC with alexithymia was found by Assogna and colleagues [73], who reported that alexithymia occurred twice as often in PD than in HC (22% vs. 11%), showing an almost four times higher risk of having alexithymia in PD than in HC. In contrast to the previous reports, this last study did not found a correlation between alexithymia and depression, and reported analyses of TAS-20 subscales showing a significant difference between groups only in the difficulty in identifying feelings (F1). The authors explained these different results based on the difference between the HC groups: outpatients in studies by Costa and colleagues and hospitalised subjects in the study by Assogna and colleagues. Finally, the authors strongly suggested that alexithymia should be considered as a nonmotor symptom of PD independent of depression. Bogdanova and Cronin-Golomb [74] found a similar dissociation between depression and alexithymia, and, in addition, between apathy and alexithymia. As expected, patients with PD reported significantly higher levels of alexithymia than the HC group, particularly in the difficulty in describing and communicating feelings (F2) and in externally oriented thinking (F3) TAS-20 subscales. In addition, these authors reported a significant association between alexithymia and the disease stage of PD, as well as alexithymia and performance on non-verbally mediated measures of executive and visuospatial function, but not on verbally mediated tasks. Lastly, in a recent case-control study, Castelli and colleagues [8] showed an higher prevalence of alexithymia both in medicated PD patients (31.6%; 12/38) and in DBS PD patients (29.6%; 8/27) than in the HC group. No significant correlation was found between TAS-20 total score and, respectively, the severity of illness and neuropsychological test scores, both for the medicated and DBS PD patients. As far as depression is concerned, a positive correlation was found between TAS-20 and BDI scores in the pre-DBS group but not in the DBS group.

Taken together, these findings seem to suggest that emotion regulation could be an important cognitive and clinical symptom of PD independent of psychiatric symptoms. Specifically, the studies that use the TAS-20 reported a general impairment in emotion regulation in PD, without an agreement on the selectivity and specificity of the impairment regarding the three TAS-20 dimensions.

Our results do not confirm this conclusion. According to our data, unless the total scores for alexithymia ranged from 34 to 70 in PD group and from 24 to 76 in HC group, the analysis on TAS-20 total score and subscales score did not have any statistical difference between the two groups. Nevertheless, the bivariate correlations on the four categories of subjects (PD and HC with and without alexithymia) and the emotional levels (TAS-20, RME, and Ekman) reported an interesting correlation between RME and Ekman scores in all the categories with the exception of HC with alexithymia, confirming the close relation among the emotional levels, in particular between emotion recognition and representation. Finally, in the overall group of patients with PD, as well as in the subgroups of patients with or without alexithymia, no correlation was found among TAS-20 scores and, respectively, dopaminergic pharmacological treatment and duration of illness.

### Depressive symptoms and visuospatial abilities

Depression is a common neuropsychiatric complication among individuals with PD [78, 79], and it is generally accepted that clinically significant depressive disturbances occur in 40–50% of these patients [80]. Accordingly, another crucial question in this domain is the correlation between depressive symptom and different levels of emotion processing examined. Although no specific correlation was found in emotion representation studies (e.g., [63, 65]), one study on emotion recognition domain reported a significant correlation between the BDI score, and Ekman total score and fear recognition [46]; however, a posteriori analyses showed no correlation between severity of depressive symptoms and grey matter parameters in the regions where the latter correlated with facial emotion recognition. It is important to highlight that in most studies within this domain, the absence of depressive symptoms was an exclusion criteria and majority of these studies were not specifically designed to test this issue.

As previously discussed, the most important correlation between emotion processing and depressive symptom emerged in emotion regulation studies. Costa and colleagues [75] reported that patients with PD having major depression were more alexithymic than those who were non-depressed, and also tended to be more alexithymic than patients with minor depression, as revealed by the overall TAS-20 score; however, no difference was found between minor depression and non-depressed groups. High scores obtained on the BDI were found to strongly predict high levels of alexithymia, indicating a strong association between alexithymia and severity of depressive symptoms in PD. These effects were also obtained when partial scores on F1 and F2 subscales were considered, although major, minor, and non-depressed patients with PD did not differ in F3 subscale score. The authors indicated the relationship between alexithymia and PD in general, and between depression and alexithymia in particular, as the relevant issues in emotion processing in this population; a low association was seen between depression and the difficulty on focusing on inner affective experience (F3). Similar results were reported by Goerlich-Dobre and colleagues [76], with a strong correlation between the BDI total score and TAS-20 total scores F1 and F2, but not with the F3 subscale score. Different conclusions were proposed by the studies that used HC control group to test this correlation [10, 73, 74], where significant associations among BDI scores and TAS-20 factors were found in both PD and HC groups. Costa and colleagues [10] showed that the correlation between the BDI and TAS-20 scores tended to be higher among HC than patients with PD, while in the PD group, the F2 subscale showed the lowest association with depressive symptoms. Both the Assogna et al. [73] and Bogdanova and Cronin-Golomb [74] studies reported a specific association between TAS-20 total score and F1 subscale, and BDI scores equally in PD and HC groups. Moreover, Bogdanova and Cronin-Golomb [74] showed that the extent of depression did not correlate with performance on neuropsychological measures of attention, executive, and visuospatial functions. Lastly, the results of Castelli and colleagues [8] previously described indirectly confirmed a dissociation between alexithymia and depression. The authors found a significant correlation between alexithymia and depression only in the medicated PD group but not in the DBS group. Accordingly, the authors suggested that depression and alexithymia could be considered two distinct but partially overlapped clinical phenomena, and that DBS of the subthalamic nucleus intervention could influence some brain circuits implicated in depression but not in alexithymia. Taken together, these studies concluded that the specific association between emotion regulation and PD is independent of depression, and alexithymia in PD is not merely the expression of depressive symptoms. Our results are in accordance with these conclusions, showing no specific correlation among emotional level and psychiatric measures, such as BDI, STAI, and Apathy Evaluation Scale.

Another important question concerning emotion processing in PD is whether visuospatial abilities could be consider as a possible mediator between cognitive status and emotional abilities in this population. This issue emerged particularly in emotion regulation studies that use TAS-20 scale. Different authors suggest an association between alexithymia and visuospatial processing alterations in patients with PD, supporting the view that the right hemisphere could be specifically involved in the modulation of some facets of alexithymia [9, 74]. Patients with PD examined by Costa and colleagues [9] displayed a neuropsychological profile mainly characterised by impaired executive functions (i.e., set-maintaining and shifting) and visuospatial abilities. Patients classified as alexithymic performed significantly worse than patients without alexithymia, and HC with or without alexithymia, on several tasks requiring the elaboration of visuospatial stimuli (i.e., freehand copying of drawings, and immediate visual memory). In particular, differently than in HC, where neuropsychological variables failed to predict alexithymia, PD performances on a visuospatial task predicted a specific manifestation of alexithymia, that is, F1 subscale of TAS-20. Bogdanova and Cronin-Golomb [74] showed similar results, where alexithymia of a PD sample was associated with performance on non-verbally mediated measures of executive and visuospatial function, but not on verbally mediated tasks.

Similar effects were found by McKinlay and colleagues [66] in emotion representation level. Using the RME task, the authors found that the cognitive status of patients with PD was a significant predictor for performance on the ToM task: in particular, 54% of the total effect of cognitive status on ToM was mediated by visuospatial abilities. Our results partially confirm this conclusion: we found a correlation in emotion recognition and representation levels, in particular, between disgust recognition and performance on Corsi’s Block-Tapping Test. In addition, performance on attention matrices significantly correlated with the RME, Ekman total score, and anger recognition.

## Conclusion

PD is a common neurodegenerative condition characterised by well-known motor symptoms, whereas the presence of cognitive non-motor symptoms, such as emotional disturbances, is still underestimated. According to Péron and colleagues [5], PD provides a useful model for studying the neural substrates of emotional processing, since the dopamine depletion in the striato-thalamo-cortical circuits, like the mesolimbic dopamine system, is thought to be involved in emotional processing. One problem emerging from studies on emotional processing is the atomising approach to emotional disturbance in patients with PD, with focus of experimental studies on one specific dimension: i.e., emotion identification, affective ToM, or alexithymia.

There were limitations to the present study that need to be acknowledged. Our relatively small sample size may limit generalizability to all patients with PD, and the neuropsychological measures used to compare PD and HC groups were limited only to MMSE and FAB. Finally, the presence of emotional disturbances in PD remains under debate [11] and the empirical investigation of affective dimension in PD is far to be conclusive, at least for what concerns the influence of the disease stage in PD group selection and, more generally, for the selection criteria of the healthy control group.

Despite the limitations of this study, here we addressed the question of whether people with PD exhibit difficulties in one or more specific dimensions of emotion processing, taking into account three different levels of analyses, that is, recognition, representation, and regulation. Comparing these emotional levels, we found a specific deficit on the emotion representation level, an overall impairment on emotion recognition, and no specific impairment in emotion regulation level. The results on emotion representation, as well as the discrepancy in the performance of patients with PD found in emotion-representation literature, could possibly result from the RME task versions used in the experimental settings. As previously mentioned, one important strength of the present work is the use of the full 36-item version of the RME to test affective ToM ability. Future research should better clarify the version of RME utilised and are encouraged to use the full version of the test in order to detect mild or sub-threshold impairments in affective ToM. The use of a shorter version of RME instead of the complete 36-item version could hamper detection of emotion-representation impairments in a neurodegenerative population.
